# Improved Physical Performance Parameters in Patients Taking Nicotinamide Mononucleotide (NMN): A Systematic Review of Randomized Control Trials

**DOI:** 10.7759/cureus.65961

**Published:** 2024-08-01

**Authors:** Jimmy Wen, Burhaan Syed, Solomon Kim, Mouhamad Shehabat, Ubaid Ansari, Daniel I Razick, Muzammil Akhtar, David Pai

**Affiliations:** 1 Physical Medicine and Rehabilitation, California Northstate University College of Medicine, Elk Grove, USA; 2 Surgery, California Northstate University College of Medicine, Elk Grove, USA; 3 Internal Medicine, California Northstate University College of Medicine, Elk Grove, USA; 4 Neurology, California Northstate University College of Medicine, Elk Grove, USA; 5 Nephrology, California Northstate University College of Medicine, Elk Grove, USA

**Keywords:** nicotinamide adenine dinucleotide, physical performance, supplement, nmn, nicotinamide mononucleotide

## Abstract

Nicotinamide adenine dinucleotide (NAD+) is essential in the proper function of many essential cellular processes in the human body. The purpose of this review is to investigate the effect of nicotinamide mononucleotide (NMN), a NAD+ precursor, on physical performance and evaluate the safety profile of supplementation. A systematic review search criteria following the guidelines from the Preferred Reporting Items for Systematic Reviews and Meta-Analyses (PRISMA) was performed in four databases for randomized controlled trials on NMN supplementation. Study variables included title, author, publication date, study year, number of patients, dosage, mean age, mean follow-up time, pre- and post-intervention reported outcomes, and rates of complications. Ten studies, including 437 patients, with a mean age of 58.0 years (35.1 to 81.1 years) and a mean follow-up time of 9.6 weeks (4 to 12 weeks) were included in this study. NMN dosages ranged from 150 to 1200 mg/day. Mean pre-intervention grip strength (two studies) and skeletal mass index (two studies) were 29.9 kilograms (kg) (range: 21.4-40.1 kg) and 7.4 kg/m^2^ (range: 6.9-7.65 kg/m^2^), respectively. Mean post-intervention grip strength and skeletal mass index were 30.5 kg (range: 21.7-41.9 kg) and 7.4 kg/m^2^ (6.8-7.64 kg/m^2^), respectively. There were no serious adverse effects observed. Moreover, of the reported side effects, they were determined to be independent of NMN supplementation. Therefore, patients taking NMN supplementation demonstrated non-significantly improved physical performance parameters. NMN is well tolerated with no serious adverse effects observed.

## Introduction and background

Nicotinamide adenine dinucleotide (NAD+) is significantly involved in the reduction of NAD to NADH (nicotinamide adenine dinucleotide) during glycolysis, the tricarboxylic acid cycle (TCA), pyruvate-lactate shunting, and the subsequent oxidation of NADH to NAD for adenosine triphosphate (ATP) production [1]. NAD serves as a co-substrate for the sirtuin family (SIRT-7), a group of NAD+-dependent deacetylases and deacylases. Additionally, it plays a crucial role in the activities of poly ADP-ribose polymerase (PARP) and cluster of differentiation 38 (CD38), both of which are involved in the synthesis and hydrolysis of cyclic ADP-ribose (cADPR) [2]. Consequently, NAD is essential for regulating various physiological processes, including metabolism, inflammation, immune response, deoxyribonucleic acid (DNA) repair, chromosomal integrity, and mitochondrial function throughout the aging process [3].

Due to the significance of NAD+ in biochemical pathways, researchers have postulated a negative correlation between NAD+ levels and aging-related diseases [4]. In animal studies, researchers have utilized nicotinamide mononucleotide (NMN), an orally bioavailable NAD+ precursor, to investigate such effects as oral administration of NAD precursors or inhibition of NAD-degrading enzymes could elevate intracellular NAD levels [4]. Although other NAD precursors are available (e.g., nicotinamide and niacin) and are commonly used to treat hyperlipidemia, they are associated with several negative side effects (e.g., flushing, hyperglycemia, and elevation of liver enzymes) [4]. Nicotinamide (NAM) and nicotinamide ribose (NR) may induce thrombocytopenia, diarrhea, nausea, skin rash, flushing, and leg cramps. Furthermore, supplementation with NAM has been associated with a significant increase in levels of uremic toxins, specifically N-methyl-nicotinamide and N-methyl-2-pyridone-5-carboxamide [5]. It is speculated that NAD upregulation can make the senescence-associated secretory phenotype (SASP) generated by senescent cells in aged tissues worse [6]. The mechanism is thought to involve the suppression of 5′ adenosine monophosphate-activated protein kinase and tumor protein p53. This suppression, in turn, leads to the activation of the nuclear factor kappa B (NF-kB) protein transcription factor through p38 mitogen-activated protein kinases [7]. Additionally, there is an observed increase in the expression of inflammatory cytokines, suggesting their potential role in this process [7].

By contrast, NMN administration has not yet illustrated such adverse effects. In these animal models, raising NAD levels through the administration of NAD precursors with NMN led to improvements in glucose and lipid metabolism, diet-induced adiposity, diabetes, diabetic kidney disease, and hepatic steatosis [8]. Reduction in vascular endothelial dysfunction and arterial stiffness, protection of the heart from ischemic damage, and increased health span further the efficacy of NMN’s efficacy in increasing NAD+ in organs, such as the heart and kidney, and mitigating aging-related diseases (e.g., obesity, kidney failure, heart failure, diabetes, stroke, and Alzheimer’s disease) [8]. However, there are some concerns surrounding NMN because many NMN products are currently sold as supplements for anti-aging and longevity. The amount of NMN present in commercially available products ranges from 50 to 150 mg per capsule; however, some individuals opt to consume two capsules of 150 mg per day [9]. Furthermore, no clear scientific evidence is available to support the safety and efficacy of these supplements. Thus, it is possible that elevating NAD+ levels unnecessarily may result in adverse physiologic effects, illustrating the need to identify the safe dose, frequency, and efficacy of NMN supplementation.

There is a paucity of studies exploring the relationship between NMN supplementation and physical performance. A six-week randomized controlled trial (RCT) involving healthy amateur athletes examined the impact of NMN oral regimens at doses of 300, 600, and 1,200 mg/day [10]. The findings indicated a significant and dose-dependent increase in aerobic capacity with exercise and NMN supplementation [10]. However, no significant effects on physical strength were observed when compared to exercise alone [10]. A different RCT spanned 12 weeks to explore the effects of a 250 mg/day oral NMN treatment on sleep quality, fatigue, and physical performance in older adults, considering the time-dependent aspects [11]. The findings indicate statistically significant interactions between physical performance, as measured by the five-times sit-to-stand test, and drowsiness [11].

An additional study explored the correlation between physical endurance, general health conditions, and NMN supplementation up to a 900 mg/day oral dose [12]. The intervention groups demonstrated the ability to walk a longer distance during the six-minute walking test compared to the placebo group [12]. Nevertheless, due to variations in demographics and dosage across studies, a systematic review is warranted to determine the most efficacious dose and the comprehensive benefits of NMN supplementation on physical performance. Further, due to a limited amount of literature available that elucidates the relationship between NMN intake and physiological effects, this systematic review investigates the efficacy of NMN in increasing physical performance in humans. The primary purpose of this review is to investigate the effect on physical performance and evaluate the safety profile of NMN supplementation. We hypothesize that NMN supplementation will have a positive effect on physical performance with minimal risk of complications.

## Review

Methods

Search Strategy

A systematic search following the Preferred Reporting Items for Systematic Reviews and Meta-Analyses (PRISMA) was conducted in PubMed, Embase, Scopus, and Cochrane Library on October 7, 2023. All authors participated in identifying the articles included in the study. The following keywords were used during the search: “nicotinamide mononucleotide,” “sport,” “athlete,” “aerobic,” “performance,” “outcome,” “longevity,” and “efficacy.”

Article Selection

Eligibility criteria and search strategy were done per the Population, Intervention, Comparison, Outcome, and Time (PICOT) framework. The patient population included patients of all ages. The intervention was NMN supplementation in this population. Comparative studies (randomized control trials) were included to compare NMN effects with placebo. The outcomes in this study were the effect of supplementation on physical performance and rates of complications. Studies with any length of follow-up were included.

The inclusion criteria focused on patients taking NMN supplements, while the exclusion criteria consisted of patients who were not taking NMN supplements. Title/abstract and full-text screening were conducted with the inclusion and exclusion criteria to determine eligibility. Articles underwent double-blinded dual-screening, with two independent reviewers screening via Covidence (Veritas Health Innovation Ltd., Melbourne, Australia). If they were not unanimous with their decisions, discrepancies were resolved with a rigorous re-review. If discrepancies persisted, a third reviewer was consulted to make the final decision on article inclusion or exclusion. All included studies underwent a thorough reference review to determine if there were additional studies to include. Finally, a manual search was performed to find additional studies that may have been missed in the original search of the two databases. This protocol is registered in the PROSPERO database as CRD42023470255.

Study Quality

The Cochrane Risk of Bias tool was utilized to determine the study quality as all studies included randomized controlled trials [13]. The tool assesses domains such as sequence generation, allocation concealment, blinding of participants and personnel, and blinding of outcome. Assessors, incomplete outcome data, selective outcome reporting, and other sources of bias were evaluated via “high,” “low,” or “unclear” risk of bias. Two authors (J.W. and B.S.) evaluated each article individually before comparing their scores. Any discrepancies were resolved by re-reviewing the articles until a consensus was reached.

Data Extraction/Analysis

Analyzed variables included title, author, publication date, study year, number of patients, mean age, mean follow-up time, dosage, pre- and post-intervention reported outcomes, and complications. All extracted data were stored and analyzed using Google Sheets (Google Drive; Google, Mountain View, CA). Descriptive statistics (mean, percentage, standard deviations, and ranges) were reported if applicable and available. A meta-analysis was intended to be performed; however, this was not possible due to the significant heterogeneity of the included studies.

Results

**Figure 1:**
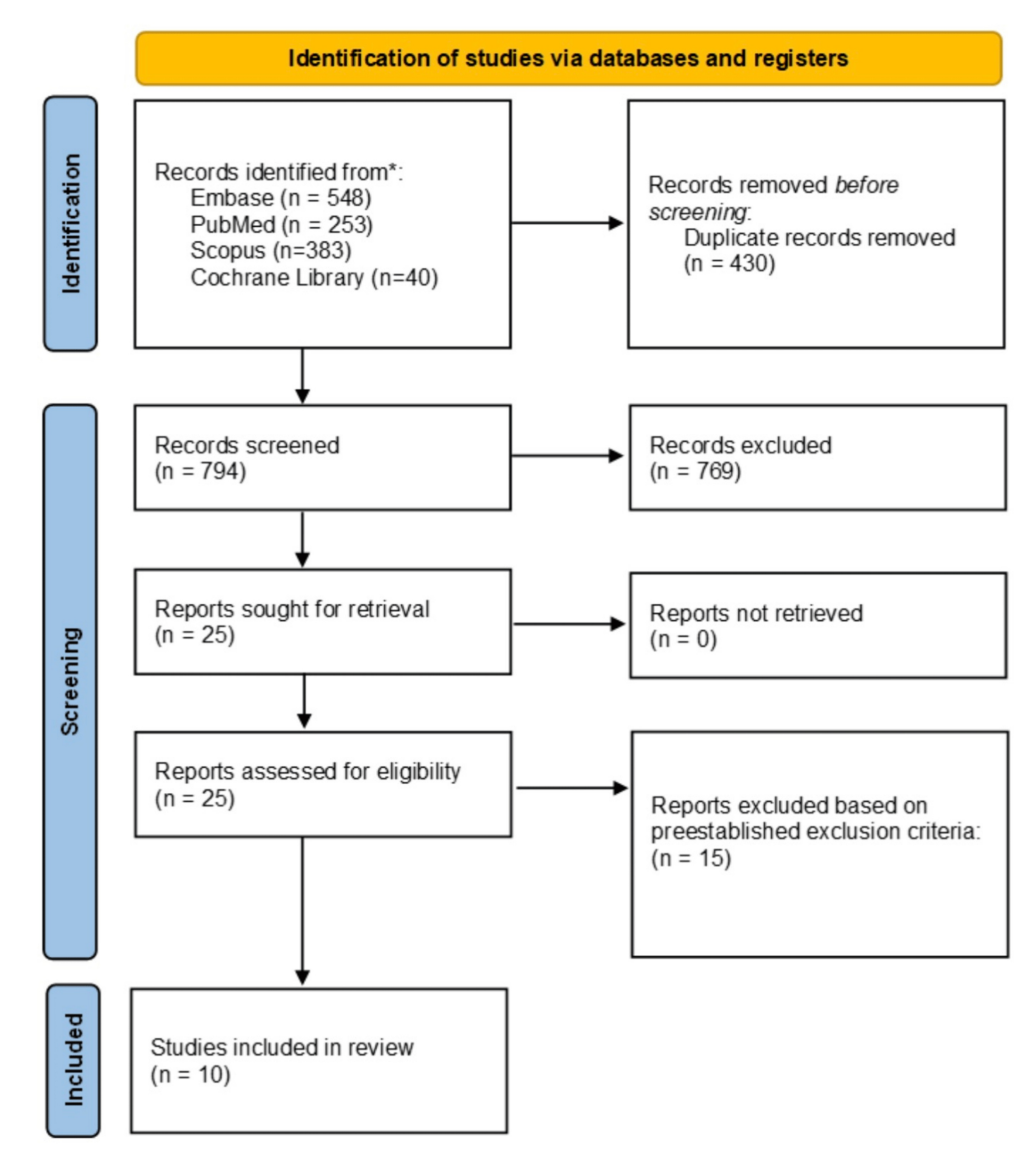
PRISMA diagram depicting the article selection process PRISMA: Preferred Reporting Items for Systematic Reviews and Meta-Analyses.

The initial search yielded 1224 records for screening. Upon removal of duplicates (n = 430) and title and abstract screening (n = 794), 25 studies remained. A full-text review led to the exclusion of 15 articles due to either being not performance-focused or wrong study design, leaving 10 total studies included in the systematic review, as shown in Figure 1.

Patient Characteristics of Included Studies

Of the 10 studies included in this systematic review, five analyzed patients above the age of 60 [11,14-17], while the remaining five focused on patients under 60 years [3,10,12,18,19]. Patient demographic data utilized in the present study included the number of patients, mean age, body mass index (BMI), and duration of follow-up. There were a total of 437 patients (48.5% male, 51.5% female), with a mean age of 58.0 years (35.1-81.1 years). The mean BMI was not reported for Akasaka et al., but for the remaining nine studies, the range was between 21.1 and 29.2 [14]. The mean follow-up time was 9.6 weeks (4-12 weeks). All 10 studies had a level of evidence (LOE) of II. Study characteristics and patient demographics are summarized in Table 1.

**Table 1:** Study characteristics and patient demographics LOE: Level of evidence; NMN: Nicotinamide mononucleotide; NR: Not reported; AM: Ante-Meridiem; PM: Post-Meridiem.

Author	Journal	Study year	LOE	Number of patients (M/F)	Age	Body mass index	Follow-up
Akasaka et al., 2023 [14]	Geriatrics and Gerontology International	Nov-22	2	NMN: 7 (7/0)	81.1 ± 6.4 years	NR	24 weeks
				Placebo: 7 (7/0)			
Connell et al., 2021 [15]	The Journal of Nutrition	Oct-21	2	14 (4/10)	72.9 ± 4.0 years	25.2 ± 2.3	32 days
								Fukamizu et al., 2022 [3]	Scientific Reports	Aug-22	2	NMN: 16 (7/9)	NMN: 35.1±7.0	NMN: 22.5 ± 3.0	4 weeks
Placebo: 15 (7/8)	Placebo: 35.7±7.2	Placebo: 22.1±3.3													
Huang, 2022 [19]	Frontiers in Aging	2022	2	NMN: 31 (13/18)	NMN: 47.76 (6.60)	NMN: 25.26 (2.34)	60 days
				Placebo: 31 (15/16)	Placebo: 47.21 (6.55)	Placebo: 24.72 (2.4)	
Igarashi et al., 2022 [16]	Nature Partner Journals Aging	May-22	2	NMN: 10 (10/0)	NMN: 71.1 ± 3.9	NMN: 24.1 ± 1.4	12 weeks
				Placebo: 10 (10/0)	Placebo: 71.8 ± 6.1	Placebo: 24.5 ± 1.4	
Kim et al., 2022 [11]	Nutrients	2022	2	NMN AM: 27 (9/18) NMN PM: 27 (9/18)	NMN AM: 72.2 ± 5.1 NMN PM: 72.8 ± 4.3	NMN AM: 22.9 ± 2.5 NMN PM: 23.4 ± 2.8	12 weeks
				Placebo AM: 27(9/18), Placebo PM: 27 (8/19)	Placebo AM: 72.5 ± 4.6, Placebo PM: 73.0 ± 4.7	Placebo AM: 22.3 ±3.2, Placebo PM: 22.4 ± 2.2	
Liao et al., 2021 [10]	Journal of International Society of Sports Nutrition	2021	2	NMN: 36 (30/6)	35.6 ± 6.1	22.0 ± 2.6	6 weeks
				Placebo: 12 (10/2)			
Okabe et al., 2022 [18]	Frontiers in Nutrition	2021	2	NMN: 15 (4/11)	NMN group: 42.9± 12.0	NMN: 21.3 ± 2.5	12 weeks
				Placebo: 15 (4/11)	Placebo group: 43.9 ± 9.9	Placebo: 21.1 ± 2.1	
Pencina et al., 2023 [17]	The Journal of Clinical Endocrinology and Metabolism	July 2019-Nov 2019	2	NMN: 21 (11/10)	61.9 ± 8.6 years	29.2 ± 3.58	28 days
				Placebo: 9 (5/4)			
Yi et al., 2023 [12]	GeroScience	May 2021-July 2021	2	60 (25/35)	NMN: 49.3	27.1	60 days
				20 (8/12)	Placebo: 46.5 ± 6.7	26.9 ± 4.9	

In general, the studies analyzed displayed a lower risk of bias for all five domains. For the first domain, there was a high risk of the sequence not being sufficiently randomly generated for two studies [11,19]. As for the second domain, there was potential bias regarding the allocation of the drug and placebo assignments for another study [15]. There was also some discrepancy in the blinding of outcome assessors due to the lack of mention of any measures used to confirm blinding [11,15,17,19]. Additionally, some studies exhibited a high likelihood of problems that could expose them to outside sources of bias [11,14-15,19], and two studies had a potential for bias from other sources [16,19]. It was determined that there was a low risk of bias for domains three, four, and five for all studies. These findings are shown in Table 2.

**Table 2:** Cochrane risk of bias assessment for the included studies

Author	Sequence generation	Allocation concealment	Blinding of participants and personnel	Blinding of outcome assessors	Incomplete outcome data	Selective outcome reporting	Other source of bias
Akasaka et al., 2023 [14]	Low	Low	Low	Low	Low	Low	High
Connell et al., 2021 [15]	Low	Unsure	Low	Unsure	Low	Low	High
Fukamizu et al., 2022 [3]	Low	Low	Low	Low	Low	Low	Low
Huang, 2022 [19]	High	Low	Low	Unsure	Low	Low	High
Igarashi et al., 2022 [16]	Low	Low	Low	Low	Low	Low	Unsure
Kim et al., 2022 [11]	Low	Low	Low	Low	Low	Low	High
Liao et al., 2021 [10]	High	Low	Low	Unsure	Low	Low	High
Okabe et al., 2022 [18]	Low	Low	Low	Low	Low	Low	Low
Pencina et al., 2023 [17]	Low	Low	Low	Unsure	Low	Low	Low
Yi et al., 2023 [12]	Low	Low	Low	Low	Low	Low	Low

Patient-Reported Outcomes for Physical Performance

The included studies (10) conducted randomized, double-blind, placebo-controlled trials. Common physical performance metrics throughout all 10 studies included grip strength [11,14,16] and change in skeletal muscle mass index [14,16]. For grip strength (two studies), the pre-intervention mean was 29.9 kg (range: 21.4-40.1 kg), and the post-intervention was 30.5 kg (range: 21.7-41.9 kg). Other unique performance parameters were also reported in each study. Several studies noted adverse events that occurred during the experiment as well, accounting for the safety of NMN supplements. NMN administration protocols also varied between the studies in terms of dosage, timing, and experiment length. Table 3 summarizes these findings.

**Table 3:** Summary of the physical parameters of the included studies mg: Milligram; kg: Kilogram; s: Second; km: Kilometer; AM: Ante-Meridiem; PM: Post-Meridiem; ∆: Change in; L: Liter; min: Minute; bpm: Beats per minute; RER: Respiratory exchange ratio; VO_2max_: Maximum oxygen consumption; Mets: Metabolic equivalents; W: Watt; HR: Heart rate; O_2_: Oxygen; VT1: Ventilatory threshold 1; VT2: Ventilatory threshold 2; WR: Work rate; m: meter; NR: Not reported; NMN: Nicotinamide mononucleotide.

Author	Intervention dose	Mean follow-up	Variable	Placebo post-intervention	Post-intervention	p-value	Adverse effects
Akasaka et al., 2023 [14]	0 vs 250 mg/day	24 weeks	Grip strength (kg)	21.7 ± 4.6	22.6 ± 3.7	0.69	None
			4-m walking time (s)	5.2 ± 2.9	4.1 ± 1.1	0.34	
			Knee extension muscle strength (kg)	20.0 ± 7.5	23.2 ± 4.1	0.34	
			Eye-opening single-leg standing time (s)	14.8 ± 14.9	9.4 ± 6.8	0.40	
			Five chair-standing time (s)	19.0 ± 14.0	16.7 ± 9.7	0.72	
Connell et al., 2021 [15]	0 vs 207.5 mg/day	32 days	RAND-36 Health Survey 1.0 Questionnaire	90	92	0.031	Cold sores (herpes labialis) that resolved (1)
Huang, 2022 [19]	0 vs 150 mg/day	60 days	RAND-36 Health Survey 1.0 Questionnaire	134.04 ± 18.06	141.36 ± 8.62	NR	Dyslipidemia (2)
			Six-minute walking (km)	0.53 ± 0.27	0.49 ± 0.02	NR	
Igarashi et al., 2022 [16]	0 vs 250 mg/day	12 Weeks	Gait speed	1.30 ± 0.22	1.60 ± 0.13	0.002	None
			30-second chair-stand test	14.0 ± 5.2	16.3 ± 3.6	0.267	
			Right-hand grip strength	37.3 ± 5.9	41.9 ± 5.6	0.090	
			Left-hand grip strength	34.1 ± 4.6	37.4 ± 5.8	0.177	
Kim et al., 2022 [11]	0 vs 250 mg/day	12 weeks	Grip strength (kg)	Placebo AM: 27.5 ± 6.4; Placebo PM: 26.8 ± 6.3	NMN AM: 28.6 ± 7.0; NMN PM: 27.2 ± 6.8	0.78	None
			5-times sit-to-stand (s)	Placebo AM: 5.3 ± 1.0; Placebo PM: 5.9 ± 1.7	NMN AM: 5.1 ± 0.7; NMN PM: 5.3 ± 1.1	0.04	
			Timed up-and-go (s)	Placebo AM: 5.0 ± 0.4; Placebo PM: 5.4 ± 1.2	NMN AM: 5.2 ± 0.8; NMN PM: 5.2 ± 0.6	0.36	
			5-m habitual walk (s)	Placebo AM: 3.2 ± 0.4; Placebo PM: 3.4 ± 0.7	NMN AM: 3.4 ± 0.5; NMN PM: 3.4 ± 0.6	0.26	
Liao et al., 2021 [10]	0 vs 300 mg/day	6 weeks	∆O_2_-pulse max (L/min/bpm)	0.73 (− 0.23, 1.68)	1.25 (0.23, 2.27)	0.56	None
			∆RER max	0.09 (0.00, 0.18)	−0.02 (− 0.07, 0.07)	0.36	
			∆ VO_2max_(L/min)	0.18 (0.02, 0.36)	0.23 (0.09, 0.37)	0.48	
			∆ Peak power (Mets)	0.72 (−0.04, 1.49)	1.05 (0.45, 1.65)	0.31	
			∆ Peak workload (W)	10.9 (−2.47, 24.24)	11.25 (−0.43, 22.93)	0.58	
			∆ HR@VT1 (bpm)	5.8(0.3, 11.4)	5.2 (1.1, 9.2)		
			∆ O_2_-pulse @VT1 (L/min/bpm)	0.55 (−0.37, 1.46)	1.17 (0.71, 1.62)	0.10	
			∆ VO_2max_@VT1(L/min)	0.17 (0.09, 0.24)	0.24 (0.18, 0.30)		
			∆ Power @AVT1 (Mets)	0.69 (0.35, 1.03)	1.06 (0.80, 1.31)		
			∆%VO_2max_@VT1(L/min)	2.1 (−0.85, 1.49)	3.5 (0.05, 7.00)	0.06	
			∆ HR@VT2 (bpm)	5.6 (1.9, 9.4)	6.7 (3.2, 10.2)	0.23	
			∆ O_2_-pulse @VT2 (L/min/bpm)	0.55 (−0.37, 1.47)	1.58 (0.95, 2.22)	0.16	
			∆ VO_2max_@VT2(L/min)	0.20 (0.03, 1.49)	0.39 (0.26, 0.52)	0.06	
			∆ Power @VT2 (Mets)	0.78 (0.07, 1.49)	1.78 (1.20, 2.35)	0.03	
			∆%VO_2max_@VT2(L/min)	1.55 (−1.75, 4.84)	6.67 (3.20, 10.10)	0.06	
			∆O2/∆WR slope (ml/min/w)	0.11 (−0.58, 0.79)	0.44 (−0.16, 1.04)	0.56	
	0 vs 600 mg/day		∆O_2_-pulse max (L/min/bpm)	0.73 (− 0.23, 1.68)	0.82 (0.07, 1.70)	0.56	
			∆RER max	0.09 (0.00, 0.18)	0.05 (− 0.16, 0.11)	0.36	
			∆ VO_2max_ (L/min)	0.18 (0.02, 0.36)	0.26 (1.74, 0.35)	0.48	
			∆ Peak power (Mets)	0.72 (−0.04, 1.49)	1.18 (0.78, 1.58)	0.31	
			∆ Peak workload (W)	10.9 (−2.47, 24.24)	13.58 (5.44, 21.7)	0.58	
			∆HR@VT1 (bpm)	5.8(0.3, 11.4)	12.8 (7.8, 17.8)		
			∆O_2_-pulse @VT1 (L/min/bpm)	0.55 (−0.37, 1.46)	0.92 (0.17, 2.20)	0.10	
			∆ VO_2max_@VT1(L/min)	0.17 (0.09, 0.24)	0.33 (0.25, 0.41)		
			∆Power @AVT1 (Mets)	0.69 (0.35, 1.03)	1.41 (1.02, 1.79)		
			∆%VO_2max_@VT1(L/min)	2.1 (−0.85, 1.49)	6.5 (3.62, 9.43)	0.06	
			∆ HR@VT2 (bpm)	5.6 (1.9, 9.4)	10.8 (4.5, 17.2)	0.23	
			∆ O_2_-pulse @VT2 (L/min/bpm)	0.55 (−0.37, 1.47)	0.50 (0.04, 1.91)	0.16	
			∆ VO_2max_@VT2(L/min)	0.20 (0.03, 1.49)	0.33 (0.22, 0.44)	0.06	
			∆ Power @VT2 (Mets)	0.78 (0.07, 1.49)	1.56 (1.00, 2.11)	0.03	
			∆%VO_2max_@VT2(L/min)	1.55 (−1.75, 4.84)	4.25 (1.28, 7.22)	0.06	
			∆ O_2_/∆WR slope (ml/min/w)	0.11 (−0.58, 0.79)	0.58 (0.01, 1.16)	0.56	
	0 vs 900 mg/day		∆ O_2_-pulse max (L/min/bpm)	0.73 (− 0.23, 1.68)	1.17 (0.32, 2.02)	0.56	
			∆ RER_max_	0.09 (0.00, 0.18)	0.03 (− 0.08, 0.14)	0.36	
			∆ VO_2max_(L/min)	0.18 (0.02, 0.36)	0.32 (0.20, 0.43)	0.48	
			∆ Peak power (Mets)	0.72 (−0.04, 1.49)	1.45 (0.86, 2.06)	0.31	
			∆ Peak workload (W)	10.9 (−2.47, 24.24)	13.93 (8.92, 18.95)	0.58	
			∆ HR@VT1 (bpm)	5.8 (0.3, 11.4)	16.0 (10.4, 21.5)		
			∆O_2_-pulse @VT1 (L/min/bpm)	0.55 (−0.37, 1.46)	2.00 (1.23, 2.77)	0.10	
			∆ VO_2max_@VT1(L/min)	0.17 (0.09, 0.24)	0.47 (0.34, 0.60)		
			∆ Power @AVT1 (Mets)	0.69 (0.35, 1.03)	2.13 (1.56, 2.69)		
			∆%VO_2max_@VT1(L/min)	2.1 (−0.85, 1.49)	10.3 (7.61,13.05)	0.06	
			∆ HR@VT2 (bpm)	5.6 (1.9, 9.4)	12.4 (4.4, 20.5)	0.23	
			∆ O_2_-pulse @VT2 (L/min/bpm)	0.55 (−0.37, 1.47)	1.67 (0.72, 2.62)	0.16	
			∆ VO_2max_@VT2(L/min)	0.20 (0.03, 1.49)	0.44 (0.29, 0.58)	0.06	
			∆ Power @VT2 (Mets)	0.78 (0.07, 1.49)	2.04 (1.34, 2.73)	0.03	
			∆%VO_2max_@VT2(L/min)	1.55 (−1.75, 4.84)	6.58 (3.57, 9.59)	0.06	
			∆O_2_/∆WR slope (ml/min/w)	0.11 (−0.58, 0.79)	0.69 (0.06, 1.45)	0.56	
Yi et al., 2023 [12]	0 vs 300 mg/day	60 days	Six-minute walking (m)	330 ± 117	380 ± 144	0.90	Placebo: skin rash (1), numbness and tingling in all extremities (1), weakness of right upper extremity (1), irrelevant talk (1), mouth ulcer (1), fever (1), NMN: hyperacidity (1), skin problem (1), mouth ulcer (1), and not related to NMN treatment.
			SF-36 (Score)	128 ± 13	137 ± 12	0.12	
	0 vs 600 mg/day		Six-minute walking (m)	330 ± 117	435 ± 104	0.079	
			SF-36 (Score)	128 ± 13	136 ± 12	0.003	
	0 vs 900 mg/day		Six-minute walking (m)	330 ± 117	480 ± 128		
			SF-36 (Score)	128 ± 13	140 ± 11		
Fukamizu et al., 2022 [3]	0 vs 1250 mg/day	4 weeks	NR				Placebo: mild loose stools (1) NMN: moderate cold (1), mild loose stools (1), acne vulgaris (1), high blood pressure (1). Determined not to be due to the NMN drug.
Pencina et al., 2023 [17]	0 vs 1000 mg/day	28 days	Chest press max resistance 1 rep (Newtons)	Exact value changes from baseline were not given in the study.		0.346	Placebo: gastrointestinal disorders (3), investigations (1), respiratory/thoracic/mediastinal disorder (1) and vascular disorders(1). NMN: cardiac disorder (1), gastrointestinal disorders (2), infections and infestations (3), musculoskeletal and connective tissue disorders (2), nervous system disorders (2), psychiatric disorders (1), respiratory/thoracic/mediastinal disorder (1), and surgical/medical procedures (1).
			Chest press repetitions to failure			0.148	
			Leg press max resistance 1 rep (Newtons)			0.072	
			Leg press repetitions to failure			0.23	
			CWR time to fatigue (min)			0.605	
			CPXT total exercise time (min)			0.600	
Okabe et al., 2022 [18]	0 vs 250 mg/day	12 weeks	NR				Placebo: gastrointestinal symptoms (1), fever, joint pain, or fatigue (2), muscle pain (2), upper respiratory tract symptoms (1), headaches (2), dry eye (1), and toothaches(1). None were caused by the placebo. NMN: gastrointestinal symptoms (3), fever, joint pain, or fatigue (6), muscle pain (2), upper respiratory tract symptoms (1), and hives (1). None were caused by NMN.

Five out of the 10 studies specifically looked at NMN supplementation and performance in the older population. In a study done by Akasaka et al., grip strength (kg), four-meter walking time, knee extension muscle strength (kg), and eye-opened single-leg standing time were measured before and after the NMN and placebo were given to their respective groups [14]. After 24 weeks, no statistically significant changes were observed between the before and after metrics as well as between the placebo and NMN groups [14].

In a study by Connell et al., the short physical performance battery (SPPB) was used to measure physical performance, with scores given for activities such as the balance test, walking speed, and chair rise test [15]. Additionally, the RAND SF-36 Health Survey 1.0 questionnaire was used to measure perceived health status [15]. None of the parameter changes were statistically significant, besides the physical function aspect of the SF-36 Health Survey 1.0 questionnaire [15]. The physical functioning aspect of the questionnaire was significantly higher for the interventional group than the control group (p = 0.031) [15].

Igarashi et al. analyzed the physical performance endpoints of walking speed, 30-second chair-stand test, and right and left grip strength [16]. At weeks 6 and 12, a significant increase in gait speed was observed in the NMN group compared to the placebo group. The NMN group also had a statistically significant increase in gait speed from baseline to week 6 compared to the placebo group. This was also seen in the 30-second chair-stand test when comparing the baseline to the week 6 values. Using a mixed-model analysis, NMN improved gait speed (p = 0.033) and left-hand grip test results (p = 0.019) significantly. No other parameter had a statistically significant change at 6 or 12 weeks of NMN administration.

Kim et al. evaluated the group and investigated the effect of time-dependent intake of NMN on sleep quality, fatigue, and physical performance [11]. A total of 108 participants were divided into four groups: NMN_AM, NMN_PM, Placebo_AM, and Placebo_PM. About 250 mg of NMN or placebo was administered daily to their respective groups for 12 weeks. The AM groups would take NMN or a placebo from when they woke up to noon, and the PM group would take whatever was assigned to them from 18:00 to bedtime. The physical performance parameters used in this study were grip strength, five-times sit-to-stand (5-STS), timed up-and-go (TUG), and 5-m habitual walk. Post hoc analysis showed that for 5-STS, there was a significant improvement from pre to post-intervention for all groups. There was also a significant change between AM and PM groups for the 5-STS (p < 0.01) and TUG (p < 0.01) activities. Additionally, there was a significant change between the placebo and the NMN groups for the 5-STS (p = 0.05). A significant interaction for the 5-STS (p = 0.04) was observed when combining the supplement and time groups.

Pencina et al. explored the performance metrics of chest press max one rep max (1-RM), chest press repetition to failure, leg press max resistance 1-RM, leg press repetition to failure, loaded stair climb power, time to fatigue, and total exercise time [17]. Although there were reported increases in stair climbing power for the NMN group compared to the placebo group, the result was not statistically significant. Additionally, no other statistically significant data was reported.

In a study done by Liao et al. to determine the effect of different doses of NMN on cardiovascular health, 48 middle-aged participants from the same running team were given different dosages of NMN [10]. There were four groups: the low dosage group (300 mg/day NMN), the medium dosage group (600 mg/day NMN), the high dosage group (1200 mg/day NMN), and the control group (placebo). A baseline was obtained; the runners underwent an exercise session five to six times each week, and then values were measured after six weeks. The performance endpoints specific to cardiovascular health in the study were VO_2_, VO_2max_, VE_max_, HR_max_, heart rate reserve (HRR), respiratory exchange ratio, O_2_-pulse, peak workload, and peak power. All of these were measured at the first and second ventilatory thresholds. The study reported no significant changes between the control and any of the NMN dosage groups for HR_max_, RER_max_, HRR, O_2_-pulse, peak power, peak workload, or VO_2max_. However, there was a significant change between the baseline and the results taken six weeks later for certain parameters, which exhibited a positive effect as the dose of NMN increased compared to the control. These parameters include VO_2_@VT1 (medium p < 0.01, high p < 0.01), %VO_2max_@VT1 (medium p = 0.04, high p = 0.01), HR@VT1 (medium p = 0.05, high p = 0.01), power@VT1 (medium p = 0.01, high p < 0.01), and power@VT2 (low p = 0.02, high p = 0.01).

In another study, which was conducted by Yi et al., 80 participants were evaluated on days 0, 30, and 60 [12]. The physical performance parameters used were a six-minute walking test and a general health assessment (SF-36). For the six-minute walking test, the 600 and 900 mg groups walked a significantly longer distance on both days 30 and 60 compared to day zero. No significant data were reported for the placebo and 300 mg group when comparing the baseline distance walked with the 30- and 60-day distance. However, when comparing the placebo with the three NMN groups, there was a significant increase in distance walked for all three NMN groups (p < 0.05) on both days 30 and 60. The 600 mg NMN group also walked a significantly farther distance on days 30 and 60 when compared to the 300 mg group. This was not found when comparing the 900 and 600 mg groups, perhaps suggesting a diminishing return effect. As for the SF-36, all three NMN groups gave significantly greater scores than the placebo group at day 60 (p < 0.01). The 600 and 900 mg groups also gave significantly greater scores than placebo at the 30-day mark (600 mg: p < 0.05, 900 mg: p < 0.001). Otherwise, there were no other statistically significant findings for the SF-36 test, which assessed the quality of life and included physical health.

Body Composition

For body composition parameters, the most commonly reported outcome was skeletal mass index, which was reported in two studies [14,16]. The pre-intervention mean was 7.4 kg/m^2^ (range: 6.9-7.65 kg/m^2^), and the post-intervention mean was 7.4 kg/m^2^ (range: 6.8-7.64 kg/m^2^). Other parameters included muscle thickness of the anterior tibialis muscle, muscle volume, basal metabolic rate, and segmental lean mass of the trunk and four limbs [14-16]. It was reported that none of these parameters changed significantly after intervention.

Adverse Effects

Adverse events that occurred in the included studies were also reported as a secondary objective to determine the safety of NMN supplementation. Although some studies reported no side effects [10-11,14,16], other studies noted specific events during their experiments. A total of 36 (8.2%) patients reported an adverse event, but none were categorized as severe. In Okabe et al., 125 mg of NMN and placebo were given twice daily to their respective groups for 12 weeks [18]. The study consisted of 30 participants, and the physical parameters reported were soft lean mass, left arm lean mass, skeletal muscle mass, and body fat percentage. None of these parameters had statistically significant changes when comparing the NMN and placebo groups. The secondary goal of this study was to determine the safety of NMN supplements in a healthy subject. Adverse events for the NMN group included a participant complaining of abdominal pain right after taking NMN, which subsequently went away after 30 minutes. Although no serious adverse event was reported, abdominal pains, upper respiratory tract symptoms, and hives were reported for the NMN group. Fever, joint pain, fatigue, and muscle pain were also reported [18].

Other studies reported several adverse effects as well; however, they were generally not considered as a result of the NMN. In the study by Connell et al., a participant reported cold sores, which resolved without intervention [15]. In Fukamizu et al., NMN patients reported high blood pressure, loose stool, common cold, and acne [3]. However, this was determined to be unrelated to NMN administration and was attributed to an unrelated cause, with all patients recovering from these symptoms [3]. Two cases of dyslipidemia were noted in Huang et al, one from each group [19]. In a study by Pencina et al., cardiac, constitutional, gastrointestinal, nervous system, psychiatric, musculoskeletal/connective tissue, and respiratory/thoracic/mediastinal disorders, along with surgical/medical procedures, were noted in an NMN group [17]. Once again, this was determined to be unrelated to NMN administration, and none of the events were classified as severe [17]. In a dose-dependent study by Yi et al., adverse events such as hyperacidity, skin problems, and mouth ulcers occurred, and these were found to be unrelated to the NMN [12]. The consensus among these studies was that NMN administration is safe and tolerable, and the adverse events that occurred were not caused by the NMN.

Discussion

This systematic review analyzed 10 randomized controlled trials, utilizing NMN supplementation and its effects on physical performance. Through the analysis of pre- and post-intervention outcomes and rates of complications, it was demonstrated that NMN positively affects physical performance and is well tolerated with no serious adverse effects.

Complications

NAD+ levels decline with increased age and is considered a hallmark of aging [20]. Thus, NAD+ supplementation may have a greater benefit in older patients. In addition, NAD+ is essential in maintaining many essential cellular processes and has, thus, been investigated as a potential therapeutic for anti-aging, metabolic disorders, degenerative disorders, and chronic conditions such as diabetes, obesity, and cardiovascular disorders [9,21,22]. A large number of available supplements have been introduced to the market, each with differing dosage levels that have not been scientifically proven to be safe or effective. Although several NAD+ precursors are available, such as nicotinamide (NAM), nicotinic acid (NA), and nicotinamide riboside, they are associated with deleterious effects in the liver, kidney, neurodegeneration, oxidative DNA damage, and worsened plasma lipid profiles in mice models [23-26]. NMN precursor is not associated with these effects but rather improves hepatocyte function, reduces oxidative stress, provides neuroprotection, and improves plasma lipid profiles [9]. However, most of these studies were conducted in vitro or using mouse models. Further studies, especially human studies, are needed to confirm these findings and to determine the safest and most effective NAD+ precursor. Irie et al. conducted a preliminary safety trial of NMN supplementation via a single oral dose of 100, 250, or 500 mg and found no serious adverse effects [27]. Huang et al. analyzed the safety of 300 mg of NMN over 60 days and found no adverse effects [19]. These findings in the literature are supported by this study, with no serious adverse effects in patients taking dosages ranging from 207.5 to 1200 mg. All 10 studies evaluated side effects, and four out of the 10 reported no side effects. Side effects were reported in five studies, but they were determined to be independent of NMN supplementation. In addition, there was no significant difference in the rates of side effects compared to placebo.

Functional Outcomes

Skeletal muscle mass and strength deteriorate with increased age, predisposing patients to a lower quality of life. This is important for older patients as their risk of sarcopenia is higher due to age-related decline and muscle atrophy [28]. This review found that NMN is not associated with increased muscle mass or improvements in body composition but does show improvements in physical strength and aerobic performance. Seven out of 10 studies evaluated physical parameter outcomes. However, the included studies had greater numerical values for the NMN group but no statistically significant differences compared to placebo. Interestingly, Connell et al. reported no difference in physical performance, but the patients felt subjective improvements, as demonstrated by a significantly higher physical functioning aspect score on the Rand-36 questionnaire [15]. Similarly, Yi et al. reported significantly higher SF-36 scores, indicating improved overall health scores [12]. Yoshino et al. found that 250 mg/day of NMN supplementation for 10 weeks had no improvement in handgrip, knee flexors, and the strength and performance of the extensors [29]. However, this may be due to the lower dose used in the study. From these results, NMN could be utilized for individuals interested in performance improvement and for older patients who are at risk for sarcopenia.

Two studies investigated the effect of dose-dependent intake of NMN and their effect on aerobic performance. Liao et al. and Yi et al. found that all dosages of NMN (low, medium, or high) for both studies had significantly improved parameters compared to baseline in VT and distance walked in a six-minute walking test, respectively [10,12]. There is also a dose-dependent relationship, with higher doses performing better than lower doses. In mice models, NMN supplementation has been shown to increase the anaerobic threshold by increasing angiogenesis, muscle capillary density, vascular endothelium function, blood flow, and oxygen levels in skeletal muscle [30,31]. Along with the positive effects of exercise on aerobic capacity, exercise combined with NMN supplementation can have a synergistic relationship [30,31].

Kim et al. analyzed the effect of time-dependent intake of NMN [11]. NMN intake had significantly higher five-time sit-to-stand and TUG tests for PM intake compared to AM intake [11]. PM intake might be associated with improved performance indirectly through improvement in sleep quality, thus reducing overall daily fatigue [32]. PM intake of melatonin and milk has been shown to improve sleep quality, but this would require further investigation into the effect of NMN on sleep quality [32].

The optimal dosage for NMN supplementation is still unclear, but there is a dose-dependent and possibly time-dependent relationship regarding physical performance improvement. There is increasing evidence that maintenance of NAD+ levels during aging is important, and NAD+ supplementation is generally safe for human consumption [33-35]. The specific formulation or form of NAD+ precursors can affect the levels of NAD+ in the body [18]. Other factors such as age, gender, diet, exercise, and genetics also affect the level of NAD+ in the body [36]. Overall, NMN supplementation is well tolerated and safe in doses of up to 1200 mg/day and results in improved physical performance measurements. Future studies should include larger size and longer follow-up randomized controlled trials to better determine the efficacy of NMN’s effect on physical performance and safety. Additionally, these trials should analyze the effect of dose-dependent and time-dependent administration. Comparative studies between NAD+ precursor compounds are also necessary to determine the most optimal NAD+ precursor.

This systematic review has several strengths. First, this is the first systematic review covering the physical performance benefits of NMN supplementation. Second, all included studies were LOE II and were randomized controlled trials and placebo-controlled, allowing optimal comparison between treatment arms.

However, these findings should be taken in the context of its limitations. First, the included studies did not factor in the daily amount of NMN that could be ingested through diet. However, NAD+ and its precursors are present in minuscule amounts in food and would not drastically affect the results [37]. Future studies should take this into account to rule out confounding factors. Second, the sample size and follow-up time for these studies were small. Third, two studies did not include values for their post-intervention-reported outcomes. This limits the ability to directly visualize the improvements before and after intervention. Fourth, the majority of patients included in this study were older patients, and the effects of NMN supplementation may not be able to be extrapolated to different populations controlling for age, gender, and individual physiological function. Fifth, considerable heterogeneity is observed in these studies' reported outcomes, which prevents direct comparisons of physical performance parameters.

## Conclusions

NMN supplementation is associated with a nonsignificant improvement in the physical strength and aerobic performance and is well tolerated with no serious adverse effects. However, body composition and muscle mass are not significantly affected. There is a dose-dependent and possibly time-dependent relationship with these benefits. However, the limited number of studies prevents a comprehensive conclusion on the benefits of NMN. Further studies are required to better elucidate the outcomes and adverse effects of NMN supplementation. Future investigations should focus on further understanding the nuances in dosage and whether certain patient groups, such as athletes, would benefit more from NMN supplementation than others.
